# Pan-Pathway Based Interaction Profiling of FDA-Approved Nucleoside and Nucleobase Analogs with Enzymes of the Human Nucleotide Metabolism

**DOI:** 10.1371/journal.pone.0037724

**Published:** 2012-05-25

**Authors:** Louise Egeblad, Martin Welin, Susanne Flodin, Susanne Gräslund, Liya Wang, Jan Balzarini, Staffan Eriksson, Pär Nordlund

**Affiliations:** 1 Department of Anatomy, Physiology and Biochemistry, Swedish University of Agricultural Sciences, Uppsala, Sweden; 2 Structural Genomics Consortium, Department of Medical Biochemistry and Biophysics, Karolinska Institutet, Stockholm, Sweden; 3 Rega Institute for Medical Research, Leuven, Belgium; 4 Department of Medical Biochemistry and Biophysics, Karolinska Institutet, Stockholm, Sweden; 5 Centre for Biomedical Structural Biology, School of Biological Sciences, Nanyang Technological University, Singapore, Singapore; University Paris Diderot-Paris 7, France

## Abstract

To identify interactions a nucleoside analog library (NAL) consisting of 45 FDA-approved nucleoside analogs was screened against 23 enzymes of the human nucleotide metabolism using a thermal shift assay. The method was validated with deoxycytidine kinase; eight interactions known from the literature were detected and five additional interactions were revealed after the addition of ATP, the second substrate. The NAL screening gave relatively few significant hits, supporting a low rate of “off target effects.” However, unexpected ligands were identified for two catabolic enzymes guanine deaminase (GDA) and uridine phosphorylase 1 (UPP1). An acyclic guanosine prodrug analog, valaciclovir, was shown to stabilize GDA to the same degree as the natural substrate, guanine, with a ΔT_agg_ around 7°C. Aciclovir, penciclovir, ganciclovir, thioguanine and mercaptopurine were also identified as ligands for GDA. The crystal structure of GDA with valaciclovir bound in the active site was determined, revealing the binding of the long unbranched chain of valaciclovir in the active site of the enzyme. Several ligands were identified for UPP1: vidarabine, an antiviral nucleoside analog, as well as trifluridine, idoxuridine, floxuridine, zidovudine, telbivudine, fluorouracil and thioguanine caused concentration-dependent stabilization of UPP1. A kinetic study of UPP1 with vidarabine revealed that vidarabine was a mixed-type competitive inhibitor with the natural substrate uridine. The unexpected ligands identified for UPP1 and GDA imply further metabolic consequences for these nucleoside analogs, which could also serve as a starting point for future drug design.

## Introduction

Nucleotide metabolism is one of the major metabolic pathways in cells. Nucleotides are not only the building blocks for DNA and RNA but also key regulators and intermediates in a wide range of cellular signalling and other metabolic processes. Nucleotides are synthesized by either the *de novo* pathways or the salvage pathways where nucleobases, nucleosides and deoxynucleosides are recycled from nutrients or from degraded DNA, RNA and nucleotides. To provide appropriate pool sizes of nucleotides in particular cellular states, nucleotide metabolism is highly regulated by feedback binding of pathway intermediates. For example, binding of effectors such as nucleoside products or intermediates in nucleotide metabolism to regulatory sites of key enzymes often provides negative feed-back regulation, but in some cases may activate these enzymes. Due to the fundamental role of nucleotides in cellular metabolism, the enzymes of nucleotide metabolism constitute important anti-proliferative targets for treatment of cancers or for immunosuppressant therapy. Also, more than half of currently approved antiviral drugs are nucleoside-based analogs [1]–[3]. Nucleoside analogs used in antiviral and anticancer chemotherapy are prodrugs which require activation by cellular enzymes to their active forms before reaching the intended target enzymes. Due to the similarity in chemical structure of natural nucleosides and nucleotides to the nucleoside analogs (NAs) used as drugs, there is a potential for cross-reactivity with enzymes along their metabolic pathways. For example, NAs could inhibit enzymes of nucleotide metabolism by binding within the active sites. Alternatively, they might also bind to regulatory sites and thus serve as inhibitors or activators. One example is gemcitabine, which in its diphosphate form binds to and inhibits ribonucleotide reductase. Other examples are fluorouracil and floxuridine, which after conversion to fluorodeoxyuridine monophosphate, inhibit thymidylate synthase via a covalent interaction [4]. These interactions are considered to be important for the therapeutic effect but these compounds can also act as polymerase chain terminators by selective depletion of nucleotide (dNTP) pools and/or upon incorporation into the nucleic acid chain.

In the present study we have addressed the possible cross-reactivity of NAs with enzymes of human nucleotide metabolism using an *in vitro* approach. Insights into novel cross-reactivity could potentially explain some toxicity of NAs. In addition the identification of novel interactions of NAs with enzymes in nucleoside metabolism could render these compounds useful as starting points for the development of novel, specific inhibitors that target these enzymes. As a basis for the *in vitro* approach, more than 30 enzymes of human nucleotide metabolism have been purified to high homogeneity at the Structural Genomics Consortium (SGC) Laboratory at the Karolinska Institute in Stockholm. Furthermore, the structures of many of these enzymes have been determined (sgc.ki.se/structures.html) [5].

The establishment of activity assays for a large number of different enzymes is very challenging. Instead, a biophysical binding assay was used to determine the interaction of the enzymes with nucleoside analogs. Thermal shift assay (TSA) was used, where binding is detected by the thermal stabilization of proteins due to interaction with the ligand. There are several potential formats for this assay including fluorescence- and light scattering-based methods [6], [7]. One advantage is that detection of protein melting temperature using these assays can be done in a high throughput format on multi-well plates and require relative small amounts of protein sample. Similar approaches have been used to screen protein kinases [8] and sulphotransferases [9] toward panels of inhibitors and substrates. In these cases, many of the compounds were already known to interact with members of the families, due to significant sequence conservation of residues in their active sites, cross-reactivates were expected.

We have designed a nucleoside analog library (NAL) containing 45 FDA (U.S. Food and Drug Administration)-approved nucleoside drugs. This library was screened against 23 selected enzymes in human nucleotide metabolism using a light scattering-based TSA [10]. In contrast to large scale TSA-based screening on protein kinases and sulphotransferases mentioned above, our enzyme collection is composed of a wide range of structural enzyme families and therefore contains a highly divergent set of active sites and effector binding sites. The TSA approach was validated by the well-characterized enzyme, deoxycytidine kinase (dCK) which is known to activate many NAs. Most of NAs did not display significant off target effects using the TSA. However, unexpected stabilizing ligands were identified for two proteins: guanine deaminase (GDA) and uridine phosphorylase 1 (UPP1), which were further analyzed by using biochemical and structural means. Together this work established TSA as a useful strategy for screening libraries against en ensemble of diverse enzymes within pathways, as well provide new information on prodrug cross reactivity, mechanism of side effects as well as rationale for future drug design targeting the nucleotide metabolism.

## Results and Discussion

### Selection of Compounds to be Included in the Nucleoside Analog Library (NAL)

A library of NAs was designed using two criteria: the compound should be approved by the FDA as a pharmaceutical and the compound should contain a natural or modified nucleobase, nucleoside or nucleotide. Given these criteria, a search was performed using Drugbank [11], [12]. A total of 47 compounds were identified and two of them, enprofylline and pentostatin, could not be readily purchased. Thus, the nucleoside analog library contained 45 compounds and is in the following referred to as the NAL, although some compounds are nucleobase and nucleotide analogs. The compounds included in NAL are listed in Table S2 with their common name and IUPAC name. These compounds are primarily used to treat different types of cancers or viral infections caused by HIV, hepatitis B, hepatitis C, herpes simplex virus type 1, herpes simplex virus type 2 and varicella zoster virus [1]–[3], [13], [14].

### Nucleotide Metabolism Enzyme Library

**Figure 1:**
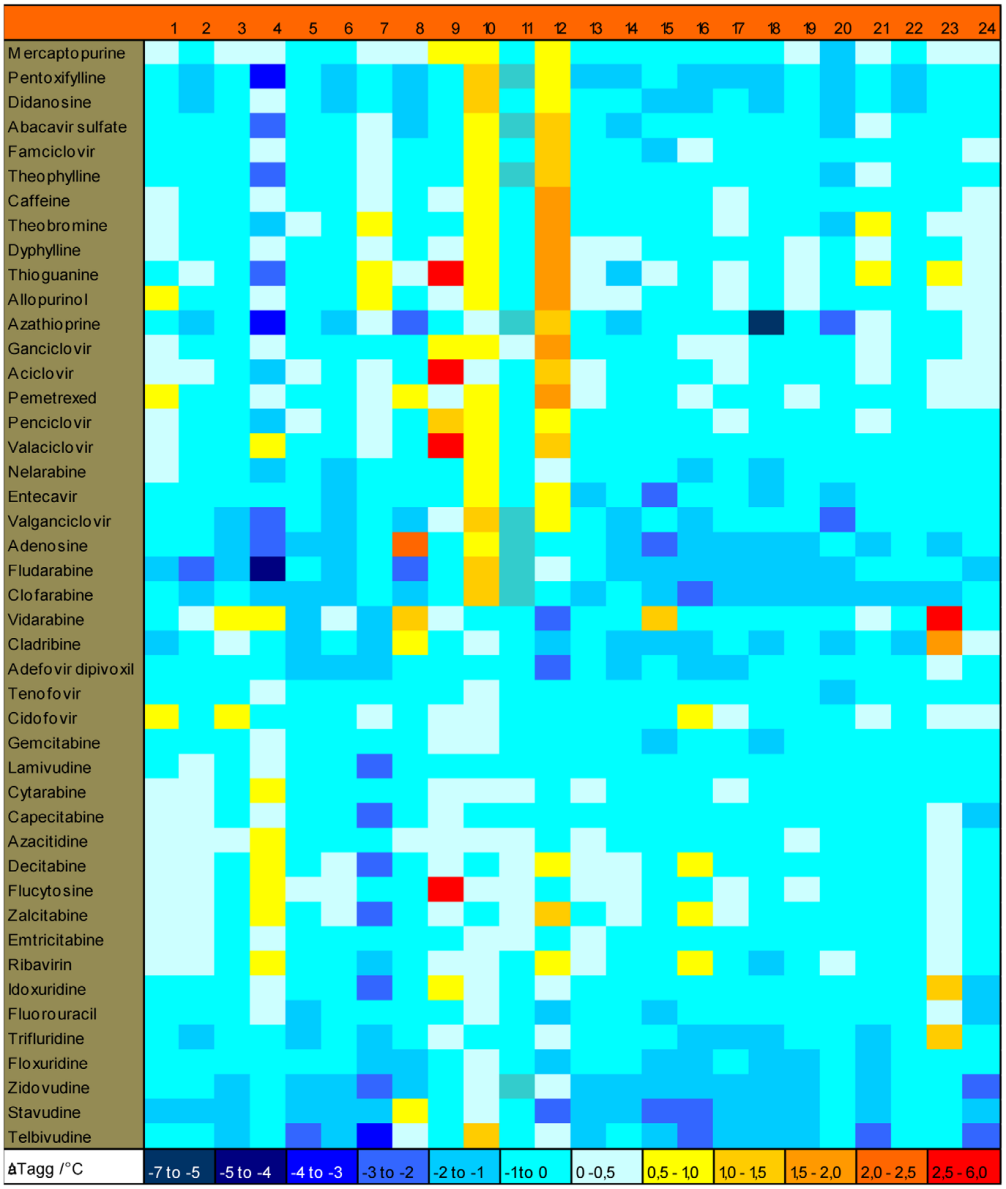
The ΔT_agg_ values for the 23 enzymes screened against NAL. The enzymes are numbered: **1** ADSS2, **2** ADSL, **3** BPNT1, **4** CMPK2, **5** CTPS2, **6** DCTD, **7** DPYS, **8** GART, **9** GDA, **10** GMPR2, **11** GMPS, **12** ITPA, **13** NT5C2, **14** NT5C3, **15** NUDT16, **16** PAICS, **17** PRTFDC1, **18** RRM1, **19** UCK1, **20** UMPS (1), **21** UMPS (2), **22** UPB1, **23** UPP1, **24** UPP2. ΔT_agg_ represents the difference between T_agg_ of a protein in the presence and absence of a compound. The ΔT_agg_ values are given as color codes based on ΔT_agg_ calculated from two values within the same screen. The maximum average mean deviation for ΔT_agg_ is 0.5°C. However, in some cases, one of the values has been disregarded due to inappropriate curve fitting parameters. In total of 1080 measurements 44 have either one value missing (8) or an average mean deviation greater than 0.5°C (36).

The proteins investigated here are listed in Table 1. They constitute enzymes of human nucleotide metabolism, for most of which expression and purification conditions have been established at the SGC-Stockholm-Karolinska Institute. Many of the proteins were also recently structurally characterized at SGC (http://www.thesgc.org/structures) [5]. Most of the proteins selected during expression and purification optimization procedure contain all their functional domains but with small truncations in the C- and N-terminus. A few of the proteins (CTPS2, RRM1, UMPS (1) and UMPS (2)) contain individual functional domains from larger multiple-domain enzymes (Table 1). Information on gene name, full enzyme name, GenBank ID, accession number, EC number, construct size, the part of nucleotide metabolism in which the enzyme is involved and T_agg_ for enzymes in the absence of ligands is given in Table 1. The enzymes are numbered, which corresponded to the number in Figure 1. However, this list of enzymes is not a complete list of all enzymes in nucleoside and nucleotide metabolism, for example, purine nucleoside phosphorylase and adenosine deaminase, which are important enzymes in the metabolism of several nucleoside and nucleobase analogs used in this study, are for technical reasons not included.

**Table 1:** List of enzymes used in this study.

Nr	Gene name	Enzyme name	GenBank ID	Accession number	EC number	Full length	Construct	Pathway	T_agg_ °C
1	ADSS2	Adenylosuccinate synthetase isozyme 2	gi|15214463	P30520	6.3.4.4	456	1–456	*De novo* purine	52.5
2	ADSL	Adenylosuccinate lyase	gi|12652985	P30566	4.3.2.2	484	1–481	*De novo* purine	61.2
3	BPNT1	3'(2'),5'-bisphosphate nucleotidase 1	gi|116812595	O95861	3.1.3.7	308	6–308	Sulfur metabolism	55.5
4	CMPK2	UMP-CMP kinase 2, mitochondrial	gi|46409274	Q5EBM0	2.7.4.14	449	27–449	*De novo* + salvage	51.6
5	CTPS2	CTP synthetase 2	gi|23271202	Q9NRF8	6.3.4.2	586	1–275	*De novo* pyrimidine	33.9
6	DCTD	Deoxycytidylate deaminase	gi|66840174	P32321	3.5.4.12	178	5–174	Catabolism pyrimidine	57.7
7	DPYS	Dihydropyrimidinase	gi|21707927	Q14117	3.5.2.2	519	4–519	Catabolism pyrimidine	56.8
8	GART	Phosphoribosylglycinamide formyltransferasePhosphoribosylamine-glycine ligase Phosphoribosylformylglycinamidine cyclo-ligase	gi|4503915	P22102	2.1.2.2	1010	1–1003	*De novo* purine	54.2
9	GDA	Guanine deaminase	gi|31566380	Q9Y2T3	6.3.4.13	1010	1–1003	*De novo* purine	
10	GMPR2	GMP reductase 2	gi|50541956	Q9P2T1	6.3.3.1	1010	1–1003	*De novo* purines	
11	GMPS	GMP synthetase	gi|4504035	P49915	3.5.4.3	454	1–454	Catabolism purine	60.1
12	ITPA	Inosine triphosphate pyrophosphatase	gi|15626999	Q9BY32	1.7.1.7	348	10–341	Catabolism purine	47.2
13	NT5C2	Cytosolic purine 5'-nucleotidase	gi|6912598	P49902	6.3.4.1/6.3.5.2	693	1–693	*De novo* purine	50.7
14	NT5C3	Cytosolic 5'-nucleotidase 3	gi|7706031	Q9H0P0	3.6.1.19	194	1–194	Catabolism	63.2
15	NUDT16	U8 snoRNA-decapping enzyme	gi|24308370	Q96DE0	3.1.3.5	561	1–561	Catabolism	56.6
16	PAICS	Phosphoribosylamino-imidazolesuccinocarboxamide synthetase Phosphoribosylaminoimidazole carboxylase	gi|16307450	P22234	3.1.3.5	336	52–336	Catabolism	51.1
17	PRTFDC1	Phosphoribosyltransferase domain containing protein 1	gi|14250450	Q9NRG1	3.6.1.30	195	1–195	Catabolism	60.3
18	RRM1	Ribonucleoside-diphosphate reductase large subunit	gi|4506749	P23921	6.3.2.6	425	1–424	*De novo* purine	64.6
19	UCK1	Uridine-cytidine kinase 1	gi|60551657	Q9HA47	4.1.1.21	425	1–424	*De novo* purine	
20	UMPS (1)	Orotate phosphoribosyltransferase	gi|4507835	P11172	2.4.2.8	225	1–225	Salvage purine	55.0
21	UMPS (2)	Orotidine-5'-phosphate decarboxylase	gi|4507835	P11172	1.17.4.1	792	75–742	*De novo* purine + pyrimidine	48.8
22	UPB1	Beta-ureidopropionase	gi|17373540	Q9UBR1	2.7.1.48	277	22–243	Salvage pyrimidine	56.4
23	UPP1	Uridine phosphorylase 1	gi|13938418	Q16831	2.4.2.10	480	7–203	*De novo* pyrimidine	42.6
24	UPP2	Uridine phosphorylase 2	gi|27597096	O95045	4.1.1.23	480	224–479	*De novo* pyrimidine	56.4

### Criteria for Thermal Shift Assay

A thermal shift assay based on light scattering was used to measure enzyme-ligand binding. Normally when proteins melt they rapidly form aggregates and this is detected by light scattering [10]. The aggregation temperature (T_agg_) measured correlates well with the melting-temperature of the proteins [15]. Binding of ligands most often increases the thermal stability e.g. the T_agg_ of the proteins [15]. Positive ΔT_agg_ values therefore indicate that a compound acted as a potential ligand for a protein. Negative ΔT_agg_ values are also sometimes observed, which indicate that a compound either destabilizes the folded protein by e.g. releasing a stronger binding ligand or binds to a protein in its unfolded state or on the path to the unfolded state. Ligands which form covalent adducts or metal-coordinated ligands may also lead to negative ΔT_agg_ values. ΔT_agg_ above 1°C may indicate specific binding of a compound to a protein and was used as initial criteria in this study [10]. For validation concentration-dependent response curves of ΔT_agg_ were established. In specific cases such as the control enzyme dCK, ΔT_agg_ below 1°C were still reporting on binding of known substrates/ligands. In the end different enzymes showed different root-mean-square deviation of the response which was used as an additional criteria for prioritizing compounds for further analysis. Compounds exhibiting a ΔT_agg_ value above 0.5°C were further examined by concentration-dependent response curve in order to validate whether they are ligands. “Fingerprints” for each enzyme screened with NAL was determined, and in many cases addition of a second known substrate or product was used to provide further complementing information [15].

### Method Validation Using dCK

dCK is an enzyme of the salvage pathway and converts deoxycytidine (dCyd), deoxyadenosine (dAdo) and deoxyguanosine (dGuo) into their corresponding monophosphates, with dCyd as the preferred substrate. Both UTP and ATP function as phosphate donors and the kinetic patterns differ depending on the phosphate donor used. The enzyme is feedback inhibited by its distal end product, deoxycytidine triphosphate (dCTP) [16]. From a clinical perspective, dCK activates several anticancer and antiviral drugs used in therapy. Among these are lamivudine, cytarabine, cladribine, gemcitabine, fludarabine, zalcitabine, vidarabine, nelarabine, decitabine, clofarabine, emtricitabine, azacytidine and didanosine [16]–[18].

dCK was tested against the NAL at two different concentrations (100 and 500 µM) in the presence and absence of 0.1 and 1 mM ATP (Table 2). Interaction between dCK and ATP (1 mM) alone gave a ΔT_agg_ value of −1.6°C.

**Table 2:** Mean ΔTagg (°C) for dCK.

NAs	500 µM NA (+1 mM ATP)	500 µM NA (+0.1 mM ATP)	500 µM NA	100 µM NA
Emtricitabine	9.11	4.21	1.65	1.23
Lamivudine	8.32	3.39	0.93	0.97
Decitabine	7.29	4.73	4.33	2.56
Clofarabine	5.45	1.66	1.68	0.37
Cladribine	5.44	1.29	−0,33	0.33
Gemcitabine	5.22	3.65	3.87	2.31
Cytarabine	4.93	2.98	2.74	1.33
Zalcitabine	0.95	−0.22	−0.61	−0.15
Entecavir	0.83	−1.29	−1.33	−0.97
Azacytidine	0.76	−0.93	−1.06	−0.32
Vidarabine	0.63	−1.02	−2.44	−0.11
Nelarabine	0.50	−2.13	−1.64	−1.14
Fludarabine	0.12	0.38	0.95*	−0.09
T_agg_	53.8	54.4	54.0	53.8

The Mean ΔT_agg_ was based on two samples within the screen. The average deviation from mean value was less than ±0.5°C. The NAs were listed according to the largest increase in thermal shifts (ΔT_agg_) in the presence of 500 µM NA and 1 mM ATP. T_agg_ for dCK in each screening is presented. * Indicate that the result is based on one value.

The compounds shown to increase the thermal stability of dCK can be divided into two groups: 1) emtricitabine, lamivudine, decitabine, clofarabine, cladribine, gemcitabine, cytarabine and fludarabine and 2) zalcitabine, entecavir, azacytidine, vidarabine and nelarabine. Interactions between dCK and group 1 ligands increased dCK thermal stability with ΔT_agg_ ≥1°C even in the absence of ATP, with cladribine as an exception. In the presence of ATP ΔT_agg_ values were significantly increased. Group 2 ligands produced mostly negative ΔT_agg_ values and only in the presence of 1 mM ATP the ΔT_agg_ values were positive, but they were still <1°C, which was the detection limit. These ligands ranked directly after the group 1 ligands in the full screen with 45 NAs, indicating that these thermal shifts, although relative small, can be assumed to be relevant. For both groups the relative shifts observed at 500 µM, either with or without ATP, were between 3 and 5°C for group 1 ligands and more than 1°C for group 2 ligands (Table 2). Thus, all compounds in Table 2 are interacting with dCK, as have previously been shown in biochemical studies [16]–[18].

Some of the interactions were further evaluated and a concentration-dependent stabilization was observed for all group 1 compounds, including cladribine, in the absence of ATP. However, in case of fludarabine a negative concentration-dependent curve was obtained, i.e. from ΔT_agg_ of 2°C at 100 µM to just below 0°C at 1000 µM (data not shown). None of the group 2 compounds exhibited positive concentration-dependent curves in the absence of ATP. The fact that the thermal shifts were greater when ATP was added, albeit the thermal shift with ATP alone was negative (at 0.5 mM ATP, ΔT_agg_ was −0.8°C and at 1 mM ATP, ΔT_agg_ was −1.6°C), indicated cooperative binding of the substrates as expected. It is interesting to note that the TSA approach allows for direct detection of interaction between dCK and both substrates, e.g. nucleoside and ATP, in spite of their transient nature, albeit it is not clear if stabilization is due to binding of a ternary complex, the products or both.

In summary, we were able to identify eight ligands (emtricitabine, lamivudine, decitabine, clofarabine, cladribine, gemcitabine, cytarabine and fludarabine) for dCK. A second set of potential ligands (zalcitabine, entecavir, azacytidine, vidarabine and nelarabine) was detected with relative thermal shifts above 1°C. All of these NAs, except for entecavir, are known to be activated by dCK. Therefore, after careful multiple measurements, a threshold (ΔT_agg_) as low as 0.5°C had allowed identification of known substrates which gave concentration dependent responses of thermal shifts for dCK.

### Screening of 23 Enzymes of the Nucleotide Metabolism Library Toward NAL

All 23 enzymes were exposed to 100 and 500 µM of the nucleoside analogs. Results are shown for the enzymes with 500 µM of the compounds (Fig. 1). Several enzymes, i.e. GART, GDA, GMPR2, ITPA, NUDT16 and UPP1, were stabilized with a ΔT_agg_ >1°C in the presence of several ligands. Destabilization (negative ΔT_agg_ value) was observed with several enzymes including CMPK2, DPYS, ITPA, NUDT16 and RRM1. It was unexpected that azathioprine caused destabilization of RRM1 with a negative ΔT_agg_ value >5°C. Therefore, Concentration-dependent (de)stabilization experiments were performed with GART, GDA, UPP1, GMPR2, ITPA and RRM1 at 100, 200, 300, 500 and 1000 µM of the nucleoside analog to monitor potential saturation effects. Significant concentration dependent effects of the ligands were observed with UPP1, GDA and RRM1 and the detailed results are described below. Both GMPR2 and ITPA showed significant shifts with many ligands but no concentration-dependent stabilization was observed. In addition ITPA, but not GMPR2, showed an unusual sensitivity to DMSO, even small variations in the DMSO concentration induced thermal shifts. Due to the lack of hits with concentration-dependent stabilization these two enzymes were not investigated further.

From the study of dCK and NAL, it is clear that addition of ATP reveals interactions with additional ligands. This approach was used in cases where the enzymes use ATP, PRPP, NADPH, ribose-1-phosphate, free phosphate ion (Pi) or pyrophosphate as a second substrate or product. PRTFDC1, UMPS (1), ITPA, NUDT16, GMPR2, UPP1, UPP2, CMPK2, CTPS2 and PAICS were all screened in the presence of a second substrate/product. A significant effect was observed only with UPP1 (see below). In the following section we will specifically describe and discuss the results obtained with UPP1, GDA and RRM1.

### Uridine Phosphorylase 1 (UPP1)

UPP1 catalyzes the reversible reaction of uridine and Pi into uracil and ribose-1-phosphate. Human UPP1 follows a steady-state ordered bi-bi kinetic mechanism. It is suggested that Pi binds to the free enzyme followed by uridine. Uracil then leaves the ternary complex, followed by dissociation of ribose-1-phosphate [19].

It is well established that UPP1 can convert fluorouracil into fluorouridine, which is subsequently phosphorylated into fluorouridine monophosphate by uridine/cytidine kinase [20], [21]. The metabolites of fluorouracil can disrupt RNA synthesis or inhibit thymidylate synthase activity, the latter which is essential for DNA synthesis and repair [21]. Toxic effects on normal tissues exerted by fluorouracil metabolites can be minimized by a high dose of uridine. One alternative approach to raise the intracellular concentrations of uridine is to inhibit UPP1 [22]. Benzylacyclouridine (BAU) is a potent inhibitor of UPP1, and has been investigated in clinical trials [23]. Calabresi et al. showed that BAU could reduce zidovudine-induced bone marrow toxicity in mice [24].

**Figure 2:**
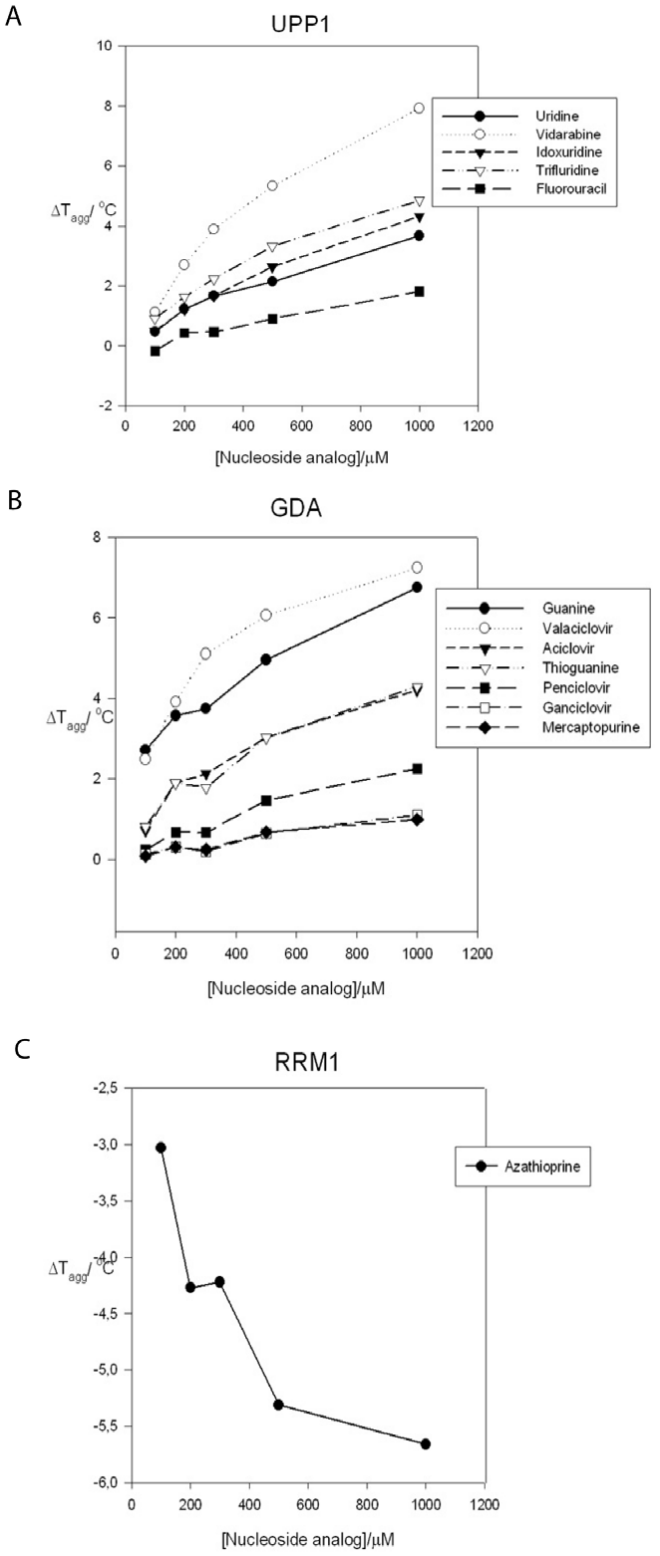
Concentration-dependent stabilization of UPP1 (A) in the presence of uridine, vidarabine, idoxuridine, trifluridine and fluorouracil; GDA (B) in the presence of guanine, valaciclovir, aciclovir, thioguanine, penciclovir, ganciclovir and mercaptopurine; and concentration dependent destabilization of RRM1 (C) in the presence of azathioprine.

**Figure 3:**
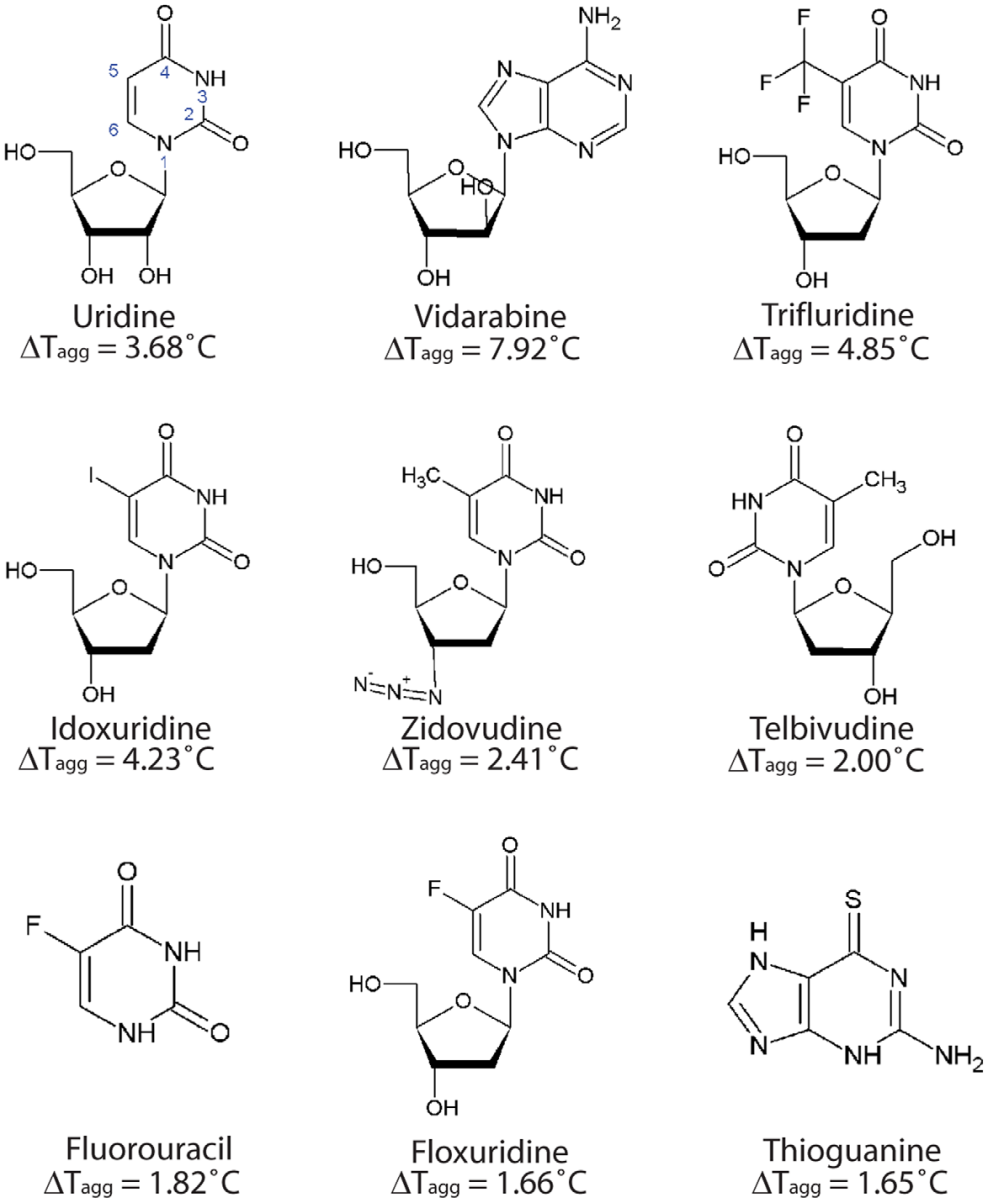
Ligands of UPP1 and mean ΔT_agg_ with 1000 µM NA.

Screening of UPP1 with 500 µM NAL revealed several potential ligands; vidarabine, trifluridine, idoxuridine, thioguanine and cladribine. Addition of ribose-1-phosphate to the protein as a second substrate identified zidovudine, fluorouracil and telbivudine as additional potential ligands. Addition of ribose-1-phosphate further stabilized UPP1 in the presence of following ligands: vidarabine, trifluridine, zidovudine and telbivudine (Table 3). To investigate saturation effects, concentration-dependent stabilization of UPP1 was tested with floxuridine, thioguanine, vidarabine, cladribine, idoxuridine, trifluridine, zidovudine, telbivudine, fluorouracil and uridine. Uridine, the natural substrate, was used as a reference. Concentration-dependent stabilization of the enzyme was found with uridine, vidarabine, trifluridine, idoxuridine and fluorouracil (Fig. 2A). In Figure 3, the structures of ligands, as well as the ΔT_agg_ values for the enzyme at 1 mM compound are shown. Vidarabine, an adenosine analog, with a ΔT_agg_ of 7.9°C at 1 mM, is the ligand that most efficiently stabilizes UPP1 and it is clearly superior to the natural substrate uridine that gave a ΔT_agg_ of 3.7°C. Idoxuridine and trifluridine stabilized the enzyme to the same extent as uridine. Concentration-dependent stabilization curves for telbivudine, zidovudine, floxuridine and thioguanine exhibited the same degree of stabilization as fluorouracil. Cladribine did not stabilize the enzyme in a concentration-dependent manner, and is therefore not regarded as a ligand.

**Table 3:** Mean ΔTagg (°C) for UPP1.

NA	ΔT_agg_ (NA+R1P)	ΔT_agg_ (NA only)
Vidarabine	6.17	4.10
Trifluridine	3.78	1.26 *
Idoxuridine	1.72	1.31 *
Thioguanine	0.86	0.65
Zidovudine	0.64	−0.39
Fluorouracil	0.59	0.42
Telbivudine	0.29	−0.52
T_agg_	56.7	57.0

The average deviation from mean value is less than ±0.5°C.

* Indicates that deviation is greater than ±0.5°C. T_agg_ of UPP1 in the absence of NA is listed. The concentration of NA was 500 µM and R1P (ribose-1-phosphate) was 1 mM.

**Figure 4:**
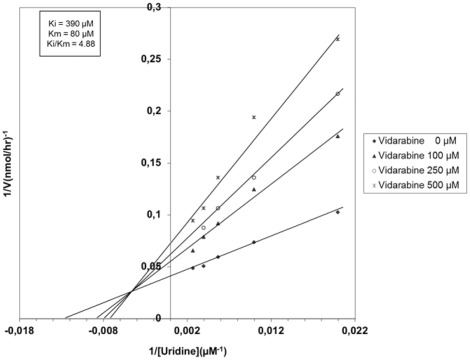
Kinetic analysis of UPP1 using Lineweaver-Burk plots. Uridine was used as substrate (50 to 375 µM) and vidarabine was as inhibitor at 500 µM (?), 250 µM (?), and 100 µM (▴) and 0 µM (⧫).

Vidarabine, the ligand stabilizing UPP1 the most; was further studied to determine the nature of vidarabine interaction with UPP1, whether it is a substrate, activator or inhibitor of the enzyme. When vidarabine was incubated with the enzyme, no conversion of vidarabine to adenine was observed by reverse phase HPLC analysis even after prolonged incubation. Therefore, a further characterization of vidarabine as an inhibitor of UPP1 was carried out. Three different vidarabine concentrations were used in the presence of five different uridine concentrations. The rates of substrate conversions to uracil as a function of uridine concentration (Fig. 4) revealed a mixed-type inhibition with a K_i_ value of 390 µM and a K_i_/K_m_ of 4.88. Thus, vidarabine have a relatively high affinity for the UPP1 enzyme. These kinetic data suggested that vidarabine binds independently of the natural substrate but altered the affinity of the enzyme for uridine, resulting in the observed mixed-type inhibition. We have not been able to determine a structure of this complex so we cannot conclude whether this is an allosteric effect or direct effect.

To our knowledge the interactions between UPP1 and the ligands revealed in this study, i.e. those involving vidarabine, idoxuridine, trifluridine, telbivudine, zidovudine and thioguanine have not been reported earlier, except for fluorouracil and floxuridine [20]. Further detailed studies of their mode of binding and effect on enzyme catalysis, such as those performed for vidarabine, should shed further light on their mode of action. Together the identification of these novel UPP1 ligands may have implications for the mechanism of activation and side effects of NAs, as well as aid in future development of nucleoside analogs.

### Guanine Deaminase (GDA)

GDA catalyzes the irreversible deamination of guanine to xanthine [25]. The enzyme is highly expressed in liver, brain, kidney and placenta [26], [27]. Furthermore, GDA is also involved in the regulation of dendrite development as a positive regulator by modulating guanine concentrations [26]. The presence of GDA activity in serum has been used as a diagnostic marker for liver disease [28]. The structure of human GDA was solved in complex with xanthine in 2007 at SGC (Pdb id: 2UZ9). Since GDA is involved in purine metabolism, as well as in the regulation of dendrite formation, it has been suggested as a potential drug target for example in the treatment of cognitive disorders [29].

**Figure 5:**
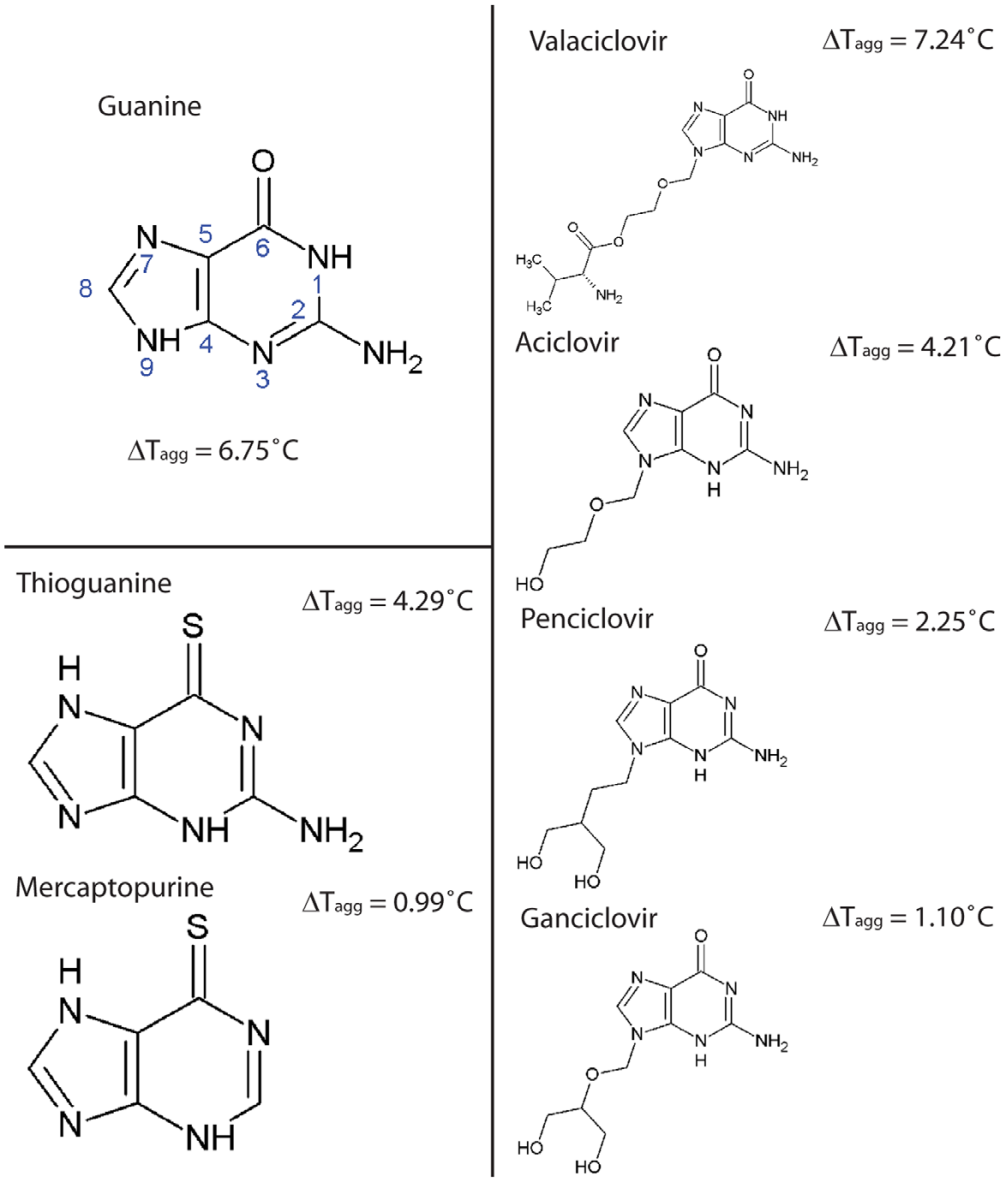
Ligands of GDA and mean ΔT_agg_ with 1000 µM NA.

All compounds exhibiting a ΔT_agg_ value greater than 0.5°C were further evaluated. Thioguanine, ganciclovir, aciclovir, penciclovir, valaciclovir, flucytosine and idoxuridine showed concentration-dependent stabilization of GDA similar to guanine (Fig. 2B), however, flucytosine and idoxuridine did not give a dose-dependent stabilization of GDA. Both valaciclovir and guanine exhibited a positive ΔT_agg_ ≈ 7°C at 1000 µM concentration, followed in decreasing order by thioguanine, acyclovir, penciclovir, ganciclovir and mercaptopurine. The chemical structures and ΔT_agg_ values of these compounds are shown in Figure 5.

**Figure 6:**
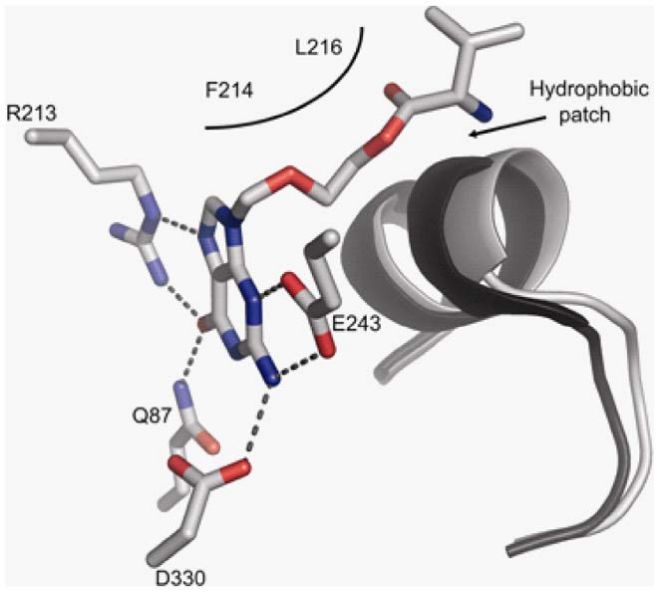
GDA in complex with valaciclovir. Amino acids that form hydrogen bonds to the guanine base of valaciclovir are shown. The α-helix containing the hydrophobic patch is superimposed with the corresponding residues of the GDA-xanthine structure (black).

In order to elucidate the molecular mechanism for the thermal shift data the structure of GDA with valaciclovir bound within the active site was determined at 2Å resolution from crystals where GDA was co-crystallized in the presence of valaciclovir (Table S1). The structure of GDA in complex with its product, xanthine, has previously been determined and is available in the Protein Data Bank (pdb-id: 2UZ9). When the GDA-xanthine structure is compared to the GDA-valaciclovir structure, no large rearrangement in the overall fold of GDA was observed. However, a hydrophobic patch (including residues Leu99, Trp102 and Leu103) in the GDA-valaciclovir structure is slightly displaced in order to accommodate the long hydrophobic tail of valaciclovir (Fig. 6). The guanine base of valaciclovir makes similar interactions as the xanthine base in the GDA-xanthine structure.

The relative ΔT_agg_ values highlight on the importance of different functional groups in these compounds. When comparing the thermal shift for thioguanine and mercaptopurine, it is evident that the loss of the amino group at the 2-position of the purine ring of mercaptopurine, leads to less stabilization of GDA. Furthermore, replacement of O with S at 6-position also decreases the binding of thioguanine and mercaptopurine to the enzyme, suggesting that the loss of hydrogen bonds between the guanine ring and Arg213 and Gln87 is the cause of weaker binding of the analogs to GDA (Fig. 6). The thermal shifts obtained with acyclic guanosine analogs suggest that both the length and hydrophobicity of the sugar mimic play an important role in binding. As demonstrated in the GDA-valaciclovir structure, the chain length of valaciclovir matched the hydrophobic path enhancing the stability of GDA, and the shorter chain length in acyclovir, penciclovir and ganciclovir were associated with smaller thermal shifts. The increased hydrophilicity of ganciclovir most likely explained the decreased thermal shifts as compared with that of penciclovir (Fig. 5).

The finding that valaciclovir and other acyclic guanosine analogs are ligands for GDA may have significant implication in the metabolism of guanine, since GDA is responsible for the catabolism of guanine and in regulation of guanine nucleotide pools. About 80% of xanthine produced in mammals occurs via guanine deamination. Valaciclovir and other acyclic guanosine analogs may act as substrates or inhibitors of GDA, and thus, affect GDA activity. Further studies are needed in order to clarify the effect of these analogs on GDA activity and the role of GDA in the metabolism of acyclic guanosine analogs.

Thioguanine, but not mercaptopurine, has previously been reported as a weak substrate for GDA [30], [31]. Whether side effects observed in treatment of various herpes virus infections could be due to altered guanine metabolism require further investigation. The knowledge gained in this study may stimulate drug development programs designed to find new substrates or inhibitors for GDA.

Fernández et al. performed *in silico* screening with human GDA (PDB id: 2UZ9) and 188 guanine analogs [32]. A number of potential ligands, including caffeine, were identified, and some of them were tested in biochemical assays using rabbit GDA as a model enzyme. Many compounds tested exhibited good inhibitory effects in the mid-micromolar range. It was suggested that longer extensions at position C2 of the purine ring would increase affinity, due to additional hydrophobic interactions [32]. In our study, caffeine had no effect on the thermal stability of human GDA. Our TSA assay and structural analysis instead demonstrated that a longer unbranched chain at position N9 of the purine base increased the thermal stability. Only a few overlaps between the list of 188 guanine analogs and our NAL are present. One example was thioguanine, which was identified as a ligand using TSA. By using *in silico* screening, this compound only ranked at position 17 out of the 188 guanine analogs [32]. An explanation for this is that the *in silico* approach do not allow for conformational modulations of the proteins upon ligand binding, whereas this might be required as exemplified by the structure of GDA-valaciclovir in this study.

### Ribonucleotide Reductase Large Subunit (RRM1)

Human ribonucleotide reductase (RR) plays an important role in the *de novo* pathway of nucleotide metabolism. RR synthesizes four deoxyribonucleoside diphosphates (dNDPs) by reducing NDPs into their dNDP forms [33]. RR in humans is composed of R1 and one of the two R2 subunits [34]. The expression of the R2 subunit is tightly regulated both at the transcriptional and post-translational levels. RR is able to maintain a balanced dNTP pool within the cell, based on its sophisticated allosteric regulation. RRM1 (name for the R1 subunit) contains three nucleotide binding sites; the catalytic site and two allosteric sites. One allosteric site is the activity site, which binds either ATP or dATP, and functions as an on-off switch. The specificity site is the other allosteric site, where ATP, dATP, dTTP or dGTP binds and alter the activity of the enzymes for the four substrates, CDP, UDP, GDP or ADP. The activity site is located in the N-terminus formed by four α-helices [33]–[35]. Since RRM1 investigated here is a truncated version, missing the first 74 amino acid residues, the activity site is absent.

No significant positive stabilization of RRM1 was seen from any member of the NAL. The destabilization of RRM1 by azathioprine, however, was concentration-dependent (Fig. 2C). We have earlier noted with other enzymes that concentration-dependent destabilization may be due to specific binding of the ligand (Larsson and Nordlund, unpublished). Azathioprine is a prodrug of mercaptopurine with an imidazole ring attached to the 6-thiol group. Mercaptopurine destabilized RRM1 also, but only to a lesser degree. Mercaptopurine-diphosphate is a substrate for ribonucleotide reductase where it is activated to the deoxy form before incorporation into DNA. It is possible that the azathioprine precursor also interacts with the enzyme although this interaction remains to be confirmed in further detail.

### Conclusions

Few cross reactivities were observed between the 23 enzymes investigated within the nucleotide pathway and the 45 FDA-approved NAs. However, we identified interactions between vidarabine, trifluridine, idoxuridine, zidovudine, telbivudine, fluorouracil, floxuridine, thioguanine and UPP1. Furthermore, vidarabine was found to be a mixed-type inhibitor of UPP1, which is suggestive of an independent binding of both the uridine substrate and the vidarabine ligand to the enzyme. Interactions were detected between GDA and valaciclovir, aciclovir, penciclovir, ganciclovir, thioguanine and mercaptopurine. UPP1 and GDA bind nucleoside and nucleobase, respectively. This is probably one reason why we were able to identify interactions with these two enzymes, since our NAL consists of both nucleosides and nucleobase analogs.

In order to make more comprehensive screen toward enzymes within the nucleotide pathway, we suggest that a full library of all enzymes in nucleoside and nucleotide metabolism and a complete library of NAs, NAMPs, NADPs and NATPs should be used. If a second substrate is necessary, as a first binder or activator, the enzyme should be screened in the presence of this, thereby obtaining the “full fingerprint” of the enzyme, as observed in case of dCK and ATP. This approach would probably generate a more extensive view of the cross-reactivities between nucleotide metabolism pathway enzymes and NAs and probably reveal additional binding of phosphorylated nucleoside adducts to these enzymes.

## Materials and Methods

### Nucleoside Analog Library

The following compounds for the NAL screening were purchased from Sigma: adefovir dipivoxil, adenosine, allopurinol, azacytidine, azathioprine, caffeine, clofarabine, cytarabine, decitabine, dyphylline, flucytosine, fluorouracil, idoxuridine, nelarabine, theobromine, theophylline, thioguanine, trifluridine, valaciclovir, vidarabine and zalcitabine. Cladribine and floxuridine were purchased from Calbiochem. Abacavir sulfate, capecitabine, famciclovir, penciclovir, pemetrexed and telbivudine were obtained from Toronto Research Chemicals. Emtricitabine, entecavir, tenofovir and valganciclovir were supplied by Moravek Biochemicals Inc. Mercaptopurine was purchased from Fischer Scientific and cidofovir was purchased from Bosche Scientific. Aciclovir, didanosine, fludarabine, ganciclovir, gemcitabine, lamivudine, pentoxyfylline, ribavirin, stavudine and zidovudine were purchased from the European Directorate for the Quality of Medicines.

### Enzyme Preparations

Genes were obtained from the National Institutes of Health Mammalian Gene Collection (for accession numbers, see Table 1) except for the dCK construct, which was a kind gift from Drs. Liya Wang and Elena Sjuvarsson, SLU, Sweden [36]. Each gene was amplified and inserted into an appropriate vector, contained either an N- or C-terminal His-tag. Vectors containing the correct insert were transformed into an E. coli expression host and stored at −80°C until further use.

Cells from glycerol stocks were inoculated into either TB or LB medium containing antibiotics and grown overnight at 37°C. Cells from overnight cultures were used to inoculate TB or LB medium (750 ml to 4.5 l) supplemented with antibiotics. Approximately 50 ml of Breox antifoam (Cognis Performance Chemical UK Ltd) was added to each flask. Cultures were grown at 37°C until an OD_600_ of 1.2 to 1.5 was obtained; this was followed by a cooling period of 1 h at 18°C in a water bath. Expression was induced by addition of 0.5 mM isopropyl-?-D-galactoside and incubation overnight at 18°C. The cells were harvested the following morning by centrifugation (5500×g, 20 min, 4°C). The resulting cell pellet was stored at −80°C.

Cells were disrupted either by sonication or high-pressure homogenization (TC5-0612W-332 from Stansted fluid power Ltd) and cell debris removed by centrifugation (49 000×g, 20 to 60 min, 4°C). The supernatant was decanted and filtered through a 0.45 µm syringe filter. Proteins were purified on an ÄKTAprime system (GE Healthcare) in a two-step process, including an IMAC Ni-charged column (1 to 5 ml HiTrap Chelating HP (GE Healthcare) and gel filtration column (Superdex 75 or 200). The IMAC column was equilibrated with IMAC wash buffer 1 (20 mM HEPES, 500 mM NaCl, 10% glycerol, 10 mM imidazole, 0.5 mM TCEP, pH 7.5). Protein was applied and washed with IMAC wash buffer 1 and 2 (20 mM HEPES, 500 mM NaCl, 10% glycerol, 25 mM imidazole, 0.5 mM TCEP, pH 7.5). Bound protein was eluted from the IMAC column with IMAC elution buffer (20 mM HEPES, 500 mM NaCl, 10% glycerol, 500 mM imidazole, 0.5 mM TCEP, pH 7.5) and automatically loaded onto the gel filtration column, which had been equilibrated with gel filtration buffer (20 mM HEPES, 300 mM NaCl, 10% glycerol, 0.5 mM TCEP, pH 7.5). Fractions were analyzed by SDS-PAGE and those containing the target proteins were pooled and concentrated using a centrifugal filter device with a 10,000 molecular weight cut off (MWCO). The identity of each protein was confirmed by mass spectrometry. Additional information can be obtained from http://www.thesgc.org/structures.

### Thermal Shift Assay Using Differential Static Light Scattering

All compounds were dissolved in 100% dimethylsulfoxide (DMSO) as 20 mM stocks, except for cidofovir which was solubilized in buffer A (20 mM HEPES pH 7.5, 300 mM NaCl, 1 mM MgCl_2_) and stored at −20°C. The compounds were diluted in buffer A at final concentrations of 100 and 500 µM in 96-well screening plates and stored at −80°C, and thawed immediately before use.

Before measuring protein aggregation using the TSA and differential static light scattering (DSLS), proteins were centrifuged for 5 min in order to remove possible protein aggregates. Protein was added to each well to a final concentration of 0.2 mg/ml (assuming a molecular weight of 35 kDa ∼ 6 µM), and transferred to 384-well optical bottom plates (#242764; Nunc, Rochester, NY, USA). The samples were run in duplicate on each screening plate. The experiments were performed using a Stargazer-384 (Harbinger Biotechnology and Engineering Corporation, Toronto, Canada) with an assay volume of 50 µl per well. 45 µl of mineral oil (#M1180, Sigma-Aldrich) was added to each well, to prevent evaporation. The plates were heated at 1°C⋅min-1, and images were taken every 0.5°C in the range 25 to 80°C. Intensities, as a measure of light scattering from protein aggregation, were converted from the images and plotted as a function of temperature. The midpoint of transition, the aggregation temperature (T_agg_) [10], [15], was calculated using the manufacturer’s software (Harbinger Biotech). ΔT_agg_ represents the calculated difference between T_agg_ of a protein in the presence of a compound and under control conditions without the compound. In some cases, proteins were screened in the presence of a co-substrate/product, which was added to the protein solution before adding it to the screening plate.

### Kinetic Studies of UPP1 with Vidarabine as Inhibitor

Reaction mixtures containing different concentrations of uridine (50, 100, 175, 250 and 375 µM), vidarabine (0, 100, 250 and 500 µM) and 1.6 ng recombinant UPP1 in a total volume of 100 µl reaction buffer (10 mM Tris–HCl pH 7.6, 300 mM NaCl, 1 mM EDTA, 2 mM KH_2_PO_4_/K_2_HPO_4_) were incubated at room temperature. After 10 min, the reaction mixtures were heated at 95°C for 3 min to inactivate the enzyme. The reaction products were separated on a reverse-phase RP-8 column (Merck Chemicals Ltd) and quantified by HPLC analysis (Alliance 2690, Waters). The separation of uridine from uracil was performed by a linear gradient from 100% separation buffer (50 mM NaH_2_PO_4_, 5 mM heptane sulfonic acid pH 3.2) to 60% separation buffer plus 40% acetonitrile (3 min 100% separation buffer; 6 min linear gradient of 100% separation buffer to 60% separation buffer plus 40% acetonitrile; 6 min 60% separation buffer plus 40% acetonitrile, followed by equilibration at 100% separation buffer). UV-based detection of uridine and uracil was performed at 253 nm and the retention time was 3.17 min (uridine), 2.50 min (uracil) and 10.15 min (vidarabine), respectively.

The data were fitted into Michealis-Menten equation using double reciprocal plots and regression lines were made for each set of data points. Mode of inhibition was deduced by Lineweaver-Burk plots.

### Crystallization, Data Collection and Structure Determination of GDA

Crystals were obtained by the sitting drop vapor diffusion method using a 96-well plate. Protein solution (15 mg/ml, 0.1 µl) containing 2 mM valaciclovir was mixed with precipitant solution (0.1 µl) containing 20% PEG 6000, 100 mM HEPES pH 7.0 and 200 mM MgCl_2_ and the drops were equilibrated at 4°C. A crystal was dipped into a cryo solution (100 mM HEPES pH 7.0, 200 mM MgCl_2_, 21% PEG 6000, 20% glycerol, 300 mM NaCl and 1.8 mM valaciclovir) and flash-frozen in liquid nitrogen. Data was collected at the Bessy beamline BL14-2 and processed with XDS [37] and Scala [38]. For molecular replacement, protein data bank (PDB) entry 2UZ9 was used as an input for MOLREP [38]. Model building and refinement were performed with COOT [39], REFMAC5 [40] and Phenix [41] (Table S1). Superpositions were made using the SSM superposition algorithm in COOT [39], [42]. Structural representations were made using PyMOL [43].

The coordinates and structure factors have been deposited to the Protein Data Bank with the accession code 4AQL. All data collection and refinement statistics are shown in Table S1.

## Supporting Information

Table S1 Data and refinement statistics for GDA in complex with valaciclovir.
(DOC)

Table S2 Common names and IUPAC names for NAs included in the NAL.
(DOC)
