# Supplementary material for: Pan-Pathway Based Interaction Profiling of FDA-Approved Nucleoside and Nucleobase Analogs with Enzymes of the Human Nucleotide Metabolism
Source: PLoS One. 2012 May 25;7(5):e37724. doi: 10.1371/journal.pone.0037724 (PMC3360617; doi:10.1371/journal.pone.0037724)
Supplement: Table S1 — Data and refinement statistics for GDA in complex with valaciclovir. (DOC) [file pone.0037724.s001.doc]

**Supporting information**

**Table S1.** Data and refinement statistics for GDA in complex with valaciclovir. Values for the highest resolution shell are shown in parentheses.

|  | **GDA-valaciclovir** |
| --- | --- |
| **Beamline** | **BESSY BL14-2** |
| **Wavelength (Å)** | **0.918** |
| **Space group** | **C222_1_** |
| **Resolution (Å)** | **33-2.0 (2.1-2.0)** |
| **Cell dimensions** |  |
| ***a*, *b*, *c* (Å)** | **86.7 91.4 131.9** |
| **R_meas_** | **0.10 (0.75)** |
| **I / σI)** | **12.4 (2.80)** |
| **Completeness (%)** | **99.5 (96.7)** |
| **Redundancy** | **4.4 (4.2)** |
| **Refinement :** |  |
| **No. reflections** | **34084** |
| **R_work_^†^/ R_free_^‡^** | **0.182 / 0.214** |
| **No. atoms** |  |
| **Protein** | **3511** |
| **Water** | **167** |
| **Other** | **24** |
| ***B*-factors (Å^2^)** |  |
| **Protein** | **25.4** |
| **Water** | **31.7** |
| **Other** | **43.8** |
| **R.m.s deviations** |  |
| **Bond lengths (Å)** | **0.013** |
| **Bond angles (°)** | **1.323** |
| **Ramachandran plot (%)^*^** |  |
| **Favoured regions** | **97.3** |
| **Additionally allowed regions** | **2.5** |

† R_work_ is defined as ΣF_obs_−F_calc_/ Σ F_obs_, where F_obs_ and F_calc_ are the observed and calculated structure-factor amplitudes, respectively.

‡ R_free_ is the R factor for the test set (5% of the data).

* According to Molprobity [44].

The coordinates and structure factors have been deposited to the Protein Data Bank with the accession code 4AQL.
