# Supplementary material for: Pan-Pathway Based Interaction Profiling of FDA-Approved Nucleoside and Nucleobase Analogs with Enzymes of the Human Nucleotide Metabolism
Source: PLoS One. 2012 May 25;7(5):e37724. doi: 10.1371/journal.pone.0037724 (PMC3360617; doi:10.1371/journal.pone.0037724)
Supplement: Table S2 — Common names and IUPAC names for NAs included in the NAL. (DOC) [file pone.0037724.s002.doc]

**Supporting information**

**Table S2.** Common names and IUPAC names for NAs included in the NAL.

| **Common name** | **IUPAC name** |
| --- | --- |
| **Mercaptopurine** | **6,7-dihydro-3H-purine-6-thione** |
| **Pentoxifylline** | **3,7-dimethyl-1-(5-oxohexyl)-2,3,6,7-tetrahydro-1H-purine-2,6-dione** |
| **Didanosine** | **9-[(2R,5S)-5-(hydroxymethyl)oxolan-2-yl]-6,9-dihydro-3H-purin-6-one** |
| **Abacavir** | **[(1S,4R)-4-[2-amino-6-(cyclopropylamino)-9H-purin-9-yl]cyclopent-2-en-1-yl]methanol** |
| **Famciclovir** | **2-[(acetyloxy)methyl]-4-(2-amino-9H-purin-9-yl)butyl acetate** |
| **Theophylline** | **1,3-dimethyl-2,3,6,7-tetrahydro-1H-purine-2,6-dione** |
| **Caffeine** | **1,3,7-trimethyl-2,3,6,7-tetrahydro-1H-purine-2,6-dione** |
| **Theobromine** | **3,7-dimethyl-2,3,6,7-tetrahydro-1H-purine-2,6-dione** |
| **Dyphylline** | **7-(2,3-dihydroxypropyl)-1,3-dimethyl-2,3,6,7-tetrahydro-1H-purine-2,6-dione** |
| **Thioguanine** | **2-amino-6,7-dihydro-3H-purine-6-thione** |
| **Allopurinol** | **1H,2H,4H-pyrazolo[3,4-d]pyrimidin-4-one** |
| **Azathioprine** | **6-[(1-methyl-4-nitro-1H-imidazol-5-yl)sulfanyl]-7H-purine** |
| **Ganciclovir** | **2-amino-9- -6,9-dihydro-3H-purin-6-one** |
| **Aciclovir or Acyclovir** | **2-amino-9-[(2-hydroxyethoxy)methyl]-6,9-dihydro-3H-purin-6-one** |
| **Pemetrexed** | **(2R)-2-{[4-(2-{2-amino-4-oxo-1H,4H,7H-pyrrolo[2,3-d]pyrimidin-5-yl}ethyl)phenyl]formamido}pentanedioic acid** |
| **Penciclovir** | **2-amino-9-[4-hydroxy-3-(hydroxymethyl)butyl]-6,9-dihydro-3H-purin-6-one** |
| **Valaciclovir or Valacyclovir** | **2-[(2-amino-6-oxo-6,9-dihydro-3H-purin-9-yl)methoxy]ethyl (2S)-2-amino-3-methylbutanoate** |
| **Nelarabine** | **(2R,3S,4S,5R)-2-(2-amino-6-methoxy-9H-purin-9-yl)-5-(hydroxymethyl)oxolane-3,4-diol** |
| **Entecavir** | **2-amino-9-[(1S,3R,4S)-4-hydroxy-3-(hydroxymethyl)-2-methylidenecyclopentyl]-6,9-dihydro-3H-purin-6-one** |
| **Valganciclovir** | **2-[(2-amino-6-oxo-6,9-dihydro-3H-purin-9-yl)methoxy]-3-hydroxypropyl (2S)-2-amino-3-methylbutanoate** |
| **Adenosine** | **(2R,3R,4S,5R)-2-(6-amino-9H-purin-9-yl)-5-(hydroxymethyl)oxolane-3,4-diol** |
| **Fludarabine** | **{[(2R,3S,4S,5R)-5-(6-amino-2-fluoro-9H-purin-9-yl)-3,4-dihydroxyoxolan-2-yl]methoxy}phosphonic acid** |
| **Clofarabine** | **(2R,3R,4S,5R)-5-(6-amino-2-chloro-9H-purin-9-yl)-4-fluoro-2-(hydroxymethyl)oxolan-3-ol** |
| **Vidarabine** | **(2R,3S,4S,5R)-2-(6-amino-9H-purin-9-yl)-5-(hydroxymethyl)oxolane-3,4-diol hydrate** |
| **Cladribine** | **(2R,3S,5R)-5-(6-amino-2-chloro-9H-purin-9-yl)-2-(hydroxymethyl)oxolan-3-ol** |
| **Adefovir dipivoxil** | **[({[2-(6-amino-9H-purin-9-yl)ethoxy]methyl}({[(2,2-dimethylpropanoyl)oxy]methoxy})phosphoryl)oxy]methyl 2,2-dimethylpropanoate** |
| **Tenofovir** | **({[(2R)-1-(6-amino-9H-purin-9-yl)propan-2-yl]oxy}methyl)phosphonic acid** |
| **Cidofovir** | **({[(2S)-1-(4-amino-2-oxo-1,2-dihydropyrimidin-1-yl)-3-hydroxypropan-2-yl]oxy}methyl)phosphonic acid** |
| **Gemcitabine** | **4-amino-1-[(2R,4R,5R)-3,3-difluoro-4-hydroxy-5-(hydroxymethyl)oxolan-2-yl]-1,2-dihydropyrimidin-2-one** |
| **Lamivudine** | **4-amino-1-[(2R,5S)-2-(hydroxymethyl)-1,3-oxathiolan-5-yl]-1,2-dihydropyrimidin-2-one** |
| **Cytarabine** | **4-amino-1-[(2R,3S,4S,5R)-3,4-dihydroxy-5-(hydroxymethyl)oxolan-2-yl]-1,2-dihydropyrimidin-2-one** |
| **Capecitabine** | **pentyl N-{1-[(2R,3R,4S,5R)-3,4-dihydroxy-5-methyloxolan-2-yl]-5-fluoro-2-oxo-1,2-dihydropyrimidin-4-yl}carbamate** |
| **Azacitidine** | **4-amino-1-[(2R,3R,4S,5R)-3,4-dihydroxy-5-(hydroxymethyl)oxolan-2-yl]-1,2-dihydro-1,3,5-triazin-2-one** |
| **Decitabine** | **4-amino-1-[(2R,4S,5R)-4-hydroxy-5-(hydroxymethyl)oxolan-2-yl]-1,2-dihydro-1,3,5-triazin-2-one** |
| **Flucytosine** | **6-amino-5-fluoro-1,2-dihydropyrimidin-2-one** |
| **Zalcitabine** | **4-amino-1-[(2R,5S)-5-(hydroxymethyl)oxolan-2-yl]-1,2-dihydropyrimidin-2-one** |
| **Emtricitabine** | **4-amino-5-fluoro-1-[(2R,5S)-2-(hydroxymethyl)-1,3-oxathiolan-5-yl]-1,2-dihydropyrimidin-2-one** |
| **Ribavirin** | **1-[(2R,3R,4S,5R)-3,4-dihydroxy-5-(hydroxymethyl)oxolan-2-yl]-1H-1,2,4-triazole-3-carboxamide** |
| **Idoxuridine** | **1-[(2R,3R,4S,5R)-3,4-dihydroxy-5-(hydroxymethyl)oxolan-2-yl]-1H-1,2,4-triazole-3-carboxamide** |
| **Fluorouracil** | **5-fluoro-1,2,3,4-tetrahydropyrimidine-2,4-dione** |
| **Trifluridine** | **1-[(2R,4S,5R)-4-hydroxy-5-(hydroxymethyl)oxolan-2-yl]-5-(trifluoromethyl)-1,2,3,4-tetrahydropyrimidine-2,4-dione** |
| **Floxuridine** | **5-fluoro-1-[(2R,4S,5R)-4-hydroxy-5-(hydroxymethyl)oxolan-2-yl]-1,2,3,4-tetrahydropyrimidine-2,4-dione** |
| **Zidovudine** | **1-[(2R,4S,5S)-4-azido-5-(hydroxymethyl)oxolan-2-yl]-5-methyl-1,2,3,4-tetrahydropyrimidine-2,4-dione** |
| **Stavudine** | **1-[(2R,5S)-5-(hydroxymethyl)-2,5-dihydrofuran-2-yl]-5-methyl-1,2,3,4-tetrahydropyrimidine-2,4-dione** |
| **Telbivudine** | **1-[(2S,4R,5S)-4-hydroxy-5-(hydroxymethyl)oxolan-2-yl]-5-methyl-1,2,3,4-tetrahydropyrimidine-2,4-dione** |
